# Principles of signal integration in combinatorial stress acclimatization

**DOI:** 10.1098/rstb.2024.0243

**Published:** 2025-05-29

**Authors:** Vijay Kumar, Madita Knieper, Lara Vogelsang, Ibadete Denjali, Thorsten Seidel, Karl-Josef Dietz

**Affiliations:** ^1^ Biochemistry and Physiology of Plants, Faculty of Biology, Bielefeld University, Bielefeld, Nordrhein-Westfalen 33615, Germany

**Keywords:** abiotic stress, signal transduction, signal integration, signalling network, hub function, evolution

## Abstract

Plants efficiently acclimatize to their environment despite experiencing varied combinations of chemical and physical parameters during their life cycle. The order of appearance, intensity and duration of relevant environmental factors, the developmental state of the plants and the cell type and organ exposed to the environmental cues add additional layers of complexity. The combinatorial diversity of environmental cues and their interaction with plants tentatively approach infinity. Beyond certain thresholds, the deviation of single or multiple environmental factors from their optimum challenges the homeostatic system to such an extent that the plants are forced into a state of stress. Given the finite complexity of genomes, response programmes to acclimatize or genetically adapt to the environment must have additional mechanisms of input integration beyond the specific detection of abiotic and biotic stress stimuli by receptors and sensors. The response to combinatorial stress parameters can be synergistic, antagonistic or indifferent and is often unpredictable from the response to single stressors. The physiological response features must have a mechanistic reflection at the molecular level. Central elements in signal processing may act as hubs of signal integration in this process, and crosstalk between signalling networks may dampen or enhance the output to the response generator system(s).

This article is part of the theme issue ‘Crops under stress: can we mitigate the impacts of climate change on agriculture and launch the ‘Resilience Revolution’?’.

## 1. The need for integrating input information to shape the acclimatization response

Plants live in an environment defined by multiple chemical and physical abiotic stimuli and biotic interactions. The response of a plant depends on its genetic capacity and epigenetic state, the ontogenetic state of development, the intensity and duration of the stresses, the order of stress impact and the previous stress history and related memory. The widely used term 'stress' lacks a reasonable definition, because neither a decreased yield nor low seed production adequately describes the previously encountered stress history. It may just be explained by (bio)chemical and physical constraints in the range of full adaptation. As an example, the biomass accumulation is lower at 15°C than at 20°C, although both temperatures may be adequate for a plant adapted to a temperate climate. An environmental condition that causes significant deviation of cell homeostasis from normality is called stress, and the redox state and reactive oxygen species (ROS) have been considered significant indicators of such stress conditions and critical signals for triggering the responses [1,2], providing the basis for quantitative stress sensing [3].

Many different signalling compounds participate in the stress response in addition to ROS—above all, plant hormones, calcium activity fluctuations and nitric oxide [4]. (i) How can these signals be decoded to initiate the optimal response tailored to acclimatize to a particular combination of external or internal inputs? (ii) What principal mechanisms are at work to integrate multiple inputs of a complex environment? (iii) What can we learn from an evolutionary consideration? (iv) Does this framework provide a mechanistic explanation as to why the acclimatory response of a biological system to combined stresses cannot be predicted from the responses to single stressors (e.g. [5–7])?

**Figure 1.**
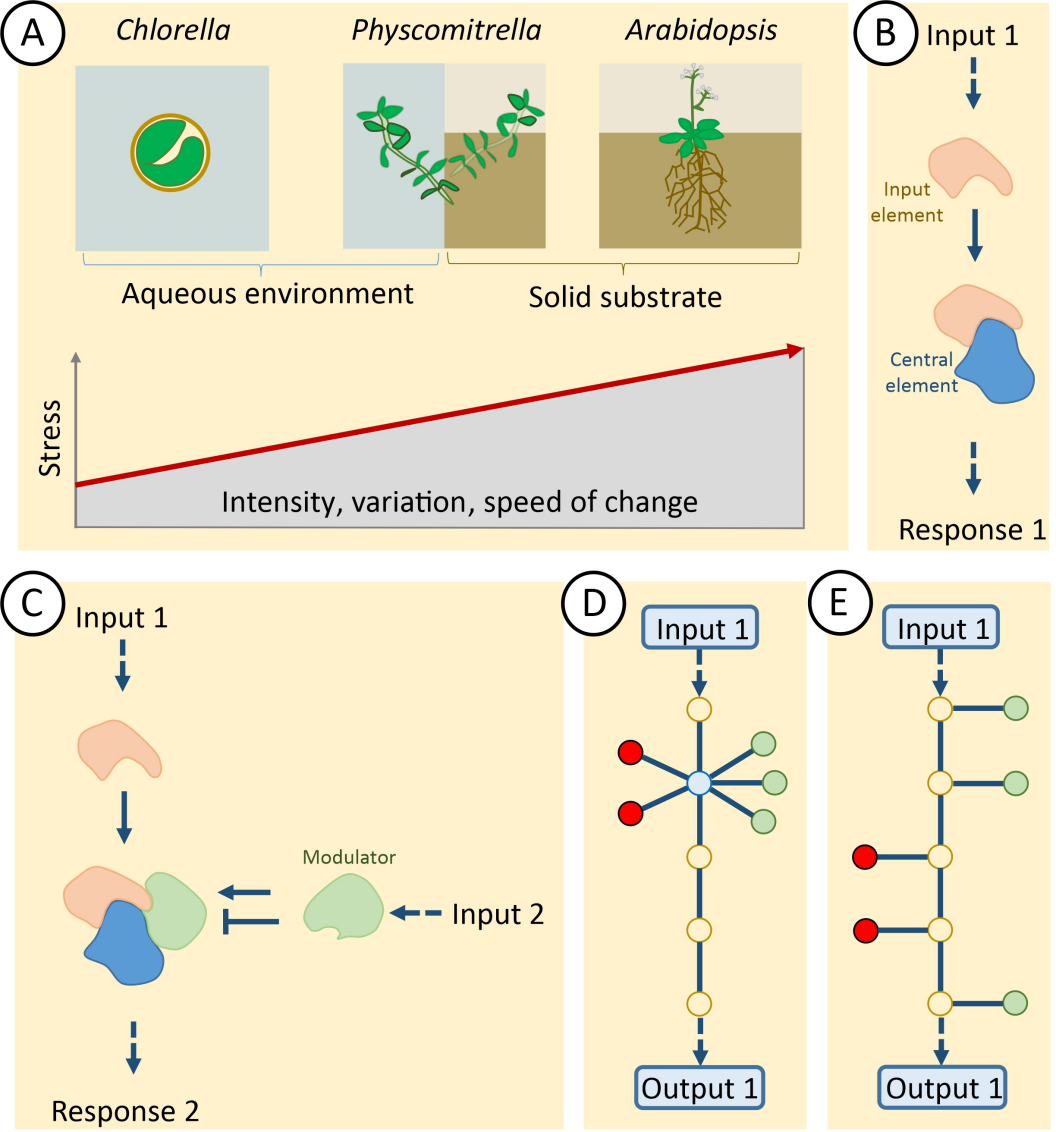
Simplified view on plant evolution and basic features of signal integration. (A) This review makes reference to three plant species ranging from the green unicellular alga *Chlorella vulgaris* to *Physcomitrella patens* as moss with first land acclimatization abilities and the model flowering plant *Arabidopsis thaliana*. The stress variability, intensity and average speed of change in nature tentatively increase along the transition from submerged growth to facultative or obligatory life on land. (B–E) Schematics on signal propagation and integration mechanisms as discussed in the text. D and E depict two scenarios, where the same modulators (red and green nodes) either act on a single signalling hub (D) or on subsequent signalling elements in a signalling pathway (E), producing either the same output 1 (as shown in this figure) or a distinct output 2. (See online version for colour references)

This review follows two concepts: first, it takes a simple evolutionary approach by comparing the unicellular alga *Chlorella vulgaris* [8] with the moss *Physcomitrella patens* [9] and the flowering plant *Arabidopsis thaliana* (figure 1A). *C. vulgaris* is a fast-growing single-celled alga with highly efficient photosynthesis. It is widely used for biotechnological applications [10]. *P. patens* grows submerged but also shows its first adaptive morphological and physiological changes to a terrestrial habitat once exposed to air. *A. thaliana* is the best-studied genetic model of a flowering plant. These three species were selected for coverage of a broad evolutionary range of photosynthetic eukaryotes. As sketched in figure 1A, the intensity, the variability and in particular the speed of variation of stress factors tentatively increase along the evolutionary transect from *C. vulgaris* via *P. patens* to *A. thaliana*.

The second concept addresses the nature of the signal integration mechanisms. At the molecular level, there exist functional entities that transmit and integrate the input signals. Since polypeptides constitute the main players in cell biology, proteins are the main actors in signal integration, whereas small molecules act as ligands. Figure 1B shows a simplified schematic where the interaction of an input element activated by an input parameter 1 affects a central regulatory element and triggers the response suitable to acclimatize to the condition represented by input 1. The scenario as shown in figure 1C emerges if an input 2 positively or negatively interferes with this signalling pathway. Here, we have a simple model for signal integration, however, cell signalling functions in the form of ‘signalling networks’, that are addressed next.

## 2. Central elements of regulation and the criteria for their function as hubs

Network theories have considered the principal structural elements of networks by graph theories. A few principal module structures allow the building of higher-order networks [11]. Basically, networks consist of nodes and edges. In our consideration, proteins form the nodes. Edges connect the nodes and represent biochemical features such as binding constants or enzyme activity like phosphorylation and dephosphorylation. The nodes also contain information on the kind of interaction effect that can be positive (= synergistic), negative (= antagonistic) or neutral (= indifferent). A primarily neutral interaction can occur with a chaperone or adaptor protein. Such an interaction does not change the activity of a protein but may enable other interactions or stabilize its state.

Graph theories have defined decisive features of networks and their elements [12]. Redundancy describes the exchangeability of one element by another. The easiest way to achieve redundancy is to express isogenes encoding related proteins with the same function. Comparison of the protein-coding capacity of the three genomes of *C. vulgare*, *P. patens* and *A. thaliana* reveals that increasing complexity of an organism often coincides with gene amplifications. In contrast, essentiality means that the deletion of an element causes a strong aberration or loss of network function. The centrality of an element increases with an increasing number of edges shared with other nodes, the so-called degree. If the degree grade equals or exceeds 5, then this element may be considered as a hub [13].

Network hubs often function in integrating input signals (figure 1D). However, similar effects may be achieved without a hub by modulating subsequent elements in a signalling pathway (figure 1E). We will elaborate on these features using three highly diverse central elements as exemplars, namely NON-EXPRESSOR OF PATHOGENESIS-RELATED GENES 1 (NPR1) involved in systemic acquired resistance (SAR) and local elicitor-triggered immunity (ETI), RESPIRATORY BURST OXIDASE HOMOLOGS (RBOHs) triggering ROS-dependent acclimatization and TARGET OF RAPAMYCIN (TOR) integrating nutritional status and growth.

### (a) NON-EXPRESSOR OF PATHOGENESIS-RELATED GENES 1 as example of signal integrator

NPR1 can be taken as a prototypic example for a central element in signal integration. NPR1 acts as a master regulator of salicylic acid (SA) induced-immune defence. It integrates input from biotic stresses (pathogen infection) and abiotic stresses (high light, freezing) by downstream adjustment of gene expression. This mechanism coordinates SAR in distal tissue and ETI in local tissue [14–16].

**Figure 2.**
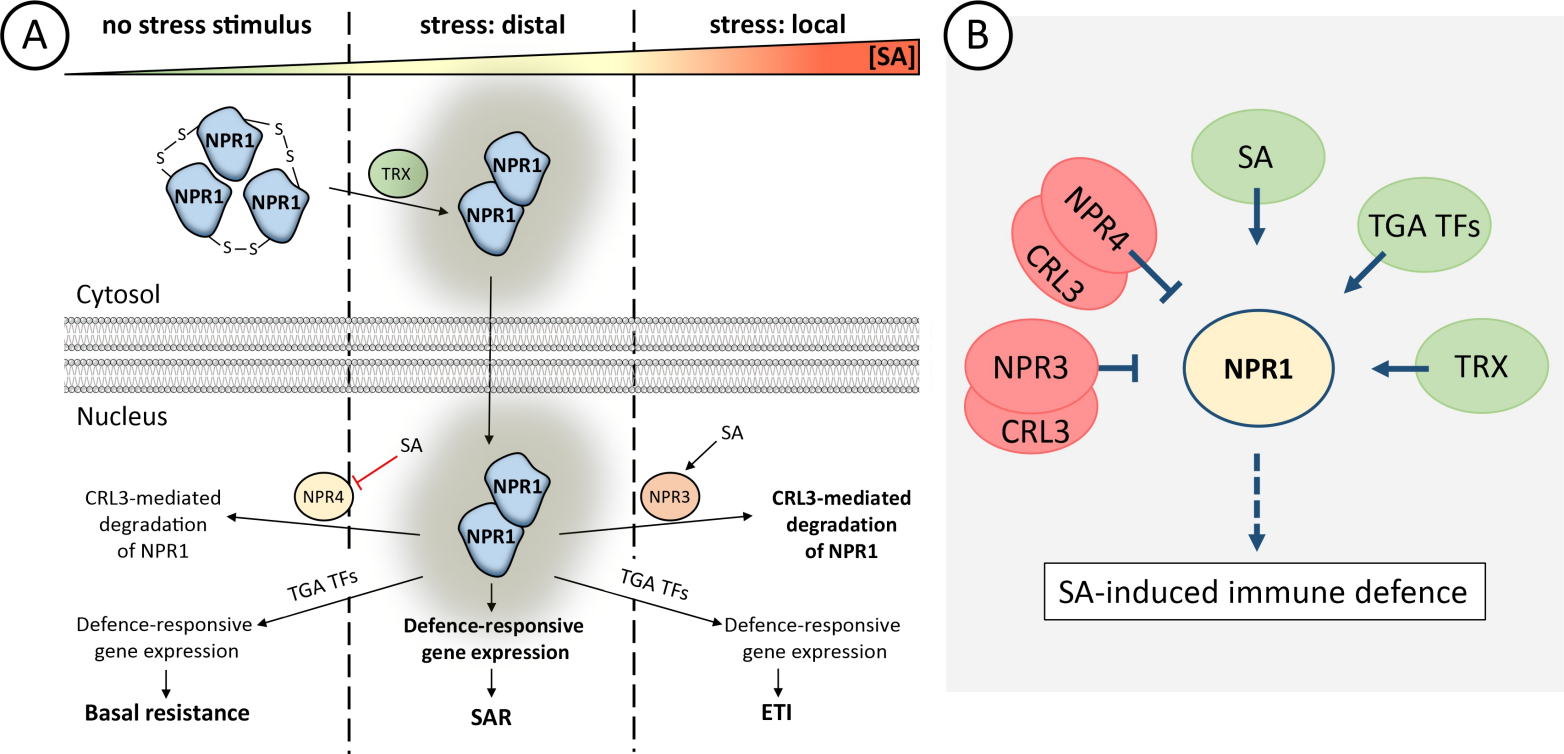
NPR-based implementation of basal resistance, systemic acquired resistance SAR and/or local elicitor-triggered immunity ETI. (A) Under optimal growth conditions, NPR1 forms oligomers in the cytosol. Minor amounts of dimeric NPR1 in the nucleus are either degraded by Cullin RING 3 Ligase - CRL3 (mediated by NPR4) or stimulate the expression of defence-related genes by interaction with TGA transcription factors. As SA concentrations rise, SA binds to NPR4, inhibiting NPR4-dependent NPR1 decay. Additionally, NPR1 oligomers are reduced and active NPR1 enters the nucleus, promoting activation of gene expression and establishing SAR. At very high concentrations, such as in local tissue after pathogen infection, SA binds to NPR3, promoting NPR3–CRL3-mediated degradation of NPR1 and establishment of ETI. (B) Simplified presentation of NPR1 as a hub model with multiple interactors, including Thioredoxin (TRX), as described in §2a.

NPR1 signalling in plant cells is controlled not only by its cellular concentration, but also by its redox state. While NPR1 forms inactive cytosolic oligomers in the absence of stress stimuli, changes in the cellular redox state under stress conditions promote thioredoxin-mediated reduction of the complex that dissociates to form monomers, or as recently suggested, homodimers (figure 2A) [17,18].

In the nucleus, these dimers interact with TGA transcription factors (bZIP TF family), activating the gene expression of stress-related genes [15,19]. Under optimal growth conditions, NPR4, which acts as a Cullin RING E3 ligase (CRL3) adaptor, mediates NPR1 degradation, preventing leaky NPR1 activity [15]. As the plant encounters stress, SA concentration in local tissue rises drastically. This enables interaction of SA with NPR3 and promotes NPR3–CRL3-catalysed NPR1 decay and subsequent induction of programmed cell death and ETI [15]. In distal tissue, where SA levels are elevated less distinctly, SA binds to NPR4 with high affinity, which disrupts NPR4-mediated NPR1 decay and results in the accumulation of nuclear NPR1 and, ultimately, SAR [15,20]. This process is further supported by SA-induced NPR1 condensates (SINCs), complexes of NPR1, CRL3 and stress proteins, in the cytosol and nucleus, which improves cell survival [21].

In *A. thaliana*, three additional homologs of NPR1 have been characterized and divided into two clades [6]. While the first clade (NPR1/2) serves as a positive regulator of defence-responsive gene expression, the second clade, comprising NPR3 and NPR4, acts as transcriptional co-repressors and antagonists of NPR1-signalling. Despite the two specialized subgroups in *A. thaliana*, the function of NPRs in plants apparently is partially conserved. For instance, the only NPR1-like protein in *P. patens* can complement NPR1-deficient *A. thaliana* lines [22]. These findings have been supported by employing NPRs from wheat, tomato, tobacco, cacao, rice and cotton in recent studies [19]. An obvious homologue is missing from the *C. vulgaris* genomes, indicating development of this regulatory module during plant lineage evolution with diversification in flowering plants.

As phytohormone signalling forms a highly complex network , NPRs not only affect SA signalling, but also the signalling of other hormones such as jasmonic acid (JA): on the one hand, NPR3 and NPR4 stimulate degradation of JAZ proteins, repressors of jasmonic acid (JA) signalling, thereby stimulating jasmonate synthesis and signalling [23]. On the other hand, interaction of NPR1 with MYC TFs in the presence of SA negatively regulates JA signalling by decreasing e.g. *LOX2* gene expression [24]. The mechanisms of regulation by NPRs completely fulfil the criteria of a hub with multiple positive and negative interacting elements, while demonstrating the intricate interplay between plant hormones (figure 2B).

### (b) The central role of RESPIRATORY BURST OXIDASE HOMOLOGS

RESPIRATORY BURST OXIDASE HOMOLOGS (RBOHs) are NADH oxidases that reside in the plasma membrane and are key players in cellular signal propagation. They reduce molecular oxygen to superoxide anion (O_2_^−^) at the apoplastic face of the plasma membrane. SUPEROXIDE DISMUTASEs (SODs) further dismutate two O_2_^−^ to one H_2_O_2_ and one O_2_. This reaction also occurs spontaneously at a low rate. H_2_O_2_ enters the cells via AQUAPORINs [25]. Through their ability to convert input signals from multiple sources to H_2_O_2_, RBOHs play a crucial role in coordinating the plant response to various stresses, including pathogen infections [26]. RBOHs carry two calcium-binding EF-hands, so that the ROS production is coupled to Ca^2+^-signalling. In addition to their role as plasma membrane-localized ROS producers, RBOHs may act together with retrograde signalling pathways from chloroplasts, mitochondria and peroxisomes to coordinate cellular responses to combinatorial stress. While the role of RBOHs in response to singular stress conditions such as pathogen attack or drought is well-documented [26,27], their role under combinatorial stress is less explored. It is a reasonable hypothesis that RBOHs integrate these signals for proper output generation by modulating ROS production in a stress-specific manner.

*A. thaliana* contains ten RBOH isoforms (A–J), which are expressed in a tissue-, development- and stress-specific manner. In a converse manner, protein blast searches detected just five RBOHs in *P. patens* and none in the unicellular *C. vulgare*, displaying the increase in complexity from green algae to mosses and higher plants and underlining the role of RBOHs in cellular signal propagation [28]. In *A. thaliana*, RBOHD is the major isoform active under conditions of abiotic and biotic stress.

**Figure 3.**
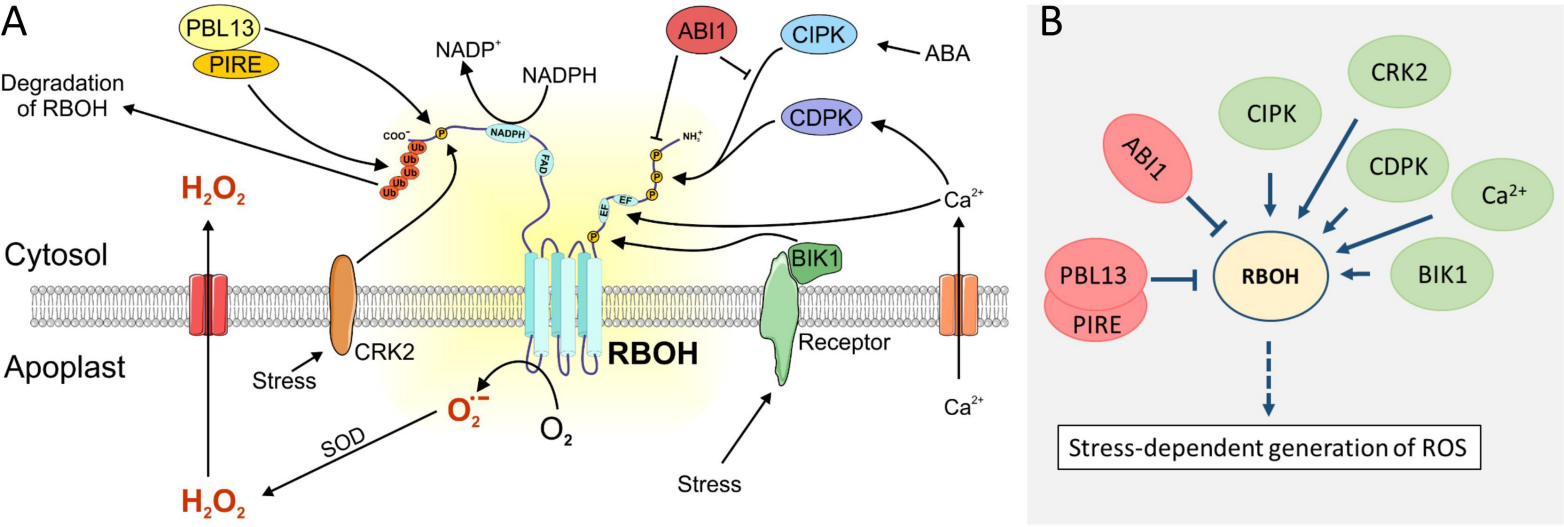
Regulation of RBOH-mediated ROS production. (A) Membrane-bound receptors sense stresses and phosphorylate RBOH either directly or indirectly via kinases like BIK1 or CDPKs at its N-terminal or C-terminal cytosolic domain to activate RBOH in presence of Ca^2+^. The phosphatase ABI1 is an antagonist of ABA-dependent phosphorylation. Inhibitory phosphorylation by PBL13 prevents activation of RBOH in the absence of stress and even results in degradation of RBOH, initiated by the ubiquitin ligase PIRE, which ubiquitinylates the C-terminus of RBOH. (B) Simplified presentation of RBOH as a hub model with multiple interactors, as described.

Next to Ca^2+^-signalling, RBOH regulation is mediated by phosphorylation of multiple phosphorylation sites at the N- and C-termini of RBOHs. For instance, Calcineurin B-like-interacting protein kinases (CIPKs), the pattern recognition receptor-associated kinase BOTRYTIS INDUCED KINASE 1 (BIK1), CYSTEINE-RICH RLK (RECEPTOR-LIKE PROTEIN KINASE) 2 (CRK2) and CALCIUM-DEPENDENT PROTEIN KINASE 16 (CDPK16) act on RBOHD in a stress-specific manner [29–31] (figure 3A). For RBOHF, it is known that ABSCISIC ACID-INSENSITIVE 1 (ABI1) functions as antagonistic phosphatase for abscisic acid (ABA)-dependent CALCINEURIN B-LIKE INTERACTING PROTEIN KINASES (CIPKs) in guard cells [32], whereas little is known about phosphatases acting on RBOHD. Phosphorylation and Ca^2+^-signalling function synergistically, possibly enhanced by 14-3-3 proteins [33]. Other regulatory factors are (i) small GTPases like ROP11, which activates RBOHF in root hairs [31], (ii) inhibitory nitrosylation of Cys890 in RBOHD [32], and (iii) inhibitory PBL13-mediated phosphorylation of the C-terminal domain followed by C-terminal ubiquitination of RBOH through the PBL13-INTERACTING RING E3 LIGASE (PIRE) and finally degradation of RBOH to prevent ROS- production in the absence of stress [34]. The complex regulation of RBOHs by various kinases, calcium signals, ubiquitination and other mechanisms underscores their central role as redox integration hubs in plant stress responses (figure 3B).

### (c) The TARGET OF RAPAMYCIN as signalling hub

In eukaryotes, the conserved TORC1 complex plays a crucial role in integrating nutrient starvation signals in order to achieve a balanced stress response by shifting growth to an acclimatized mode [35]. This regulation is further demonstrated through its reciprocal interaction with ABA, where modulation of TOR kinase activity allows plants to adapt [36]. In the plant genomes of *C. vulgaris*, *P. patens* and *A. thaliana*, the TOR complex 1 (TORC1) is encoded by the catalytic TOR subunit and functional regulatory subunits RAPTOR and LST8 [35,37]. In *A. thaliana*, the genome encodes one TOR kinase subunit gene, two RAPTOR genes, namely RAPTOR1A and RAPTOR1B, and two LST8 genes, LST8-1 and LST8-2 [29]. In algae, TOR complex subunits are encoded by one TOR kinase subunit gene, one RAPTOR and one LST8 gene, and in mosses by four RAPTOR orthologues and one LST8 orthologue, respectively [37,38]. This regulatory hub is positioned downstream of nutrient sensor components and can be positively or negatively regulated, primarily through differential stabilization or dissociation of the RAPTOR 1B subunit [39]. For example, during nitrogen (N) starvation, TOR is downregulated in both *Chlorella sorokiniana* and *A. thaliana* [40,41]. In *A. thaliana*, it has been shown that under low nitrogen conditions, the inhibition of the TORC1 complex is mediated by convergent ROP2–TOR signalling pathways, resulting in reduced leaf growth [42].

**Figure 4.**
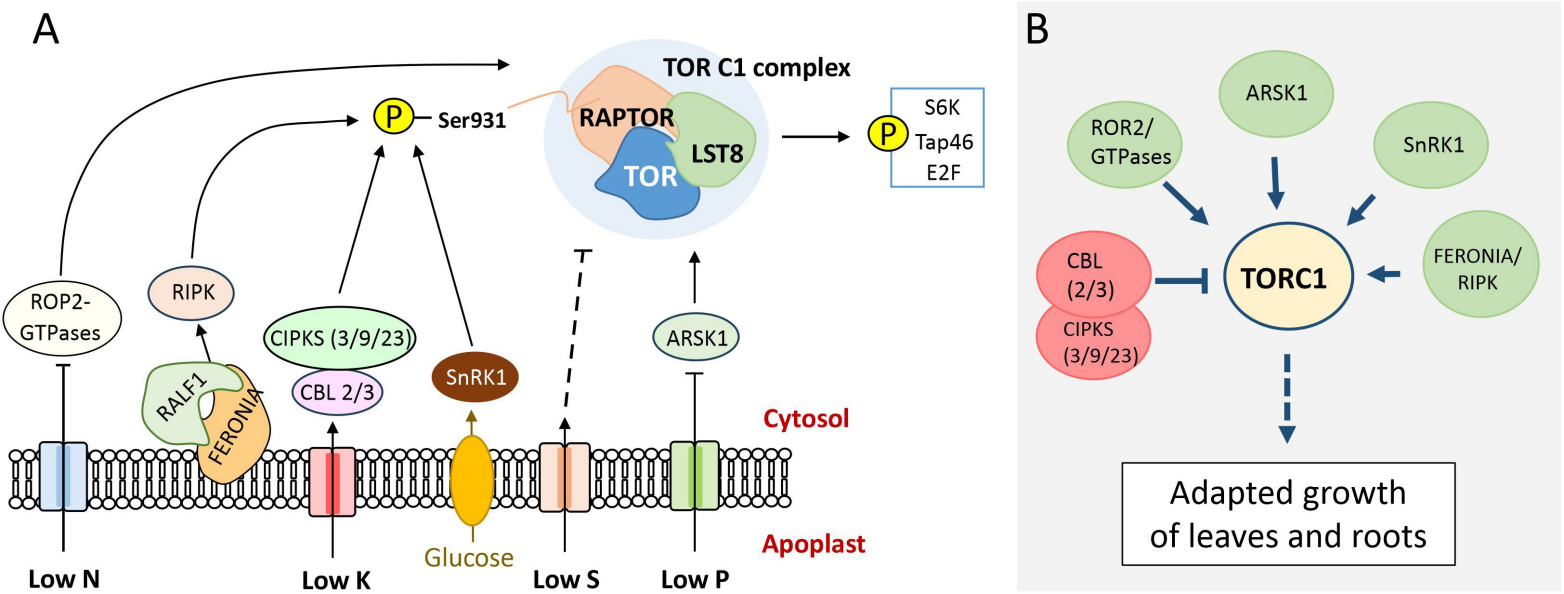
Nutrient-dependent growth and development in plants are regulated by the TORC1 complex. Under low nutrient availability, upstream components like ROP2-GTPase, FERONIA/RALF1-RIPK, CBL-CIPKs, SnRK1 and ARSK1 regulate TOR activity by phosphorylating RAPTOR1B, with the signal further relayed through phosphorylation by downstream integrators such as S6K, Tap46 and E2F. (B) Simplified presentation of TOR as a hub model with multiple interactions as described.

However, plants have evolved adaptive mechanisms to maintain growth despite low-nutrient conditions. One such adaptive mechanism involves the FERONIA (FER)/RIPK receptor kinase complex (figure 4A). Under low nitrogen conditions, this complex interacts with the RALF1 ligand to activate the TOR complex via positive phosphorylation of the RAPTOR 1B subunit, enabling sustained leaf growth under nutrient-limited conditions [43]. Furthermore, in *A. thaliana* double mutants (*cad2 sir1−1*), where the sulphur pathway is altered by blocking the sulphur flux from cysteine to glutathione, sulphur signalling through TOR regulation was revealed, leading to a shift from stress response to plant growth [44]. Further, genome transcript data indicate a connection between glucose-dependent TOR signalling pathways and the expression of sulphur assimilation genes [45].

In a converse manner, under phosphorus (P) starvation, root growth adjustments are linked to the negative regulation of ARABIDOPSIS ROOT-SPECIFIC KINASE 1 (ARSK1), which acts as an upstream inhibitor of the TOR pathway [46]. Similarly, under low potassium conditions in *A. thaliana*, CIPKs repress the TORC1 complex by triggering the dissociation of the RAPTOR 1B subunit [47] (figure 4A), providing further evidence for the interaction of TOR with several protein kinases under various stress conditions [48]. The comparison of these mechanistic findings with the discussed network theory defines the TOR complex as a hub of signal integration in complex environmental acclimatization (figure 4B).

### (d) Input interactors and modulators

Summarizing schematics of research studies, reviews and textbook pathways often depict linear transduction pathways starting from an input parameter and ending with an output. The degree of interconnectedness of nodes represents branching of the pathway. But as described before, multiple interactions with input interactors and modulators (as well as with output interactors, which are not discussed further here) characterize the function of the hub and its regulation. Calmodulin-like proteins were selected for exemplifying specialization and multiplication of input interactors and modulators during evolution.

### (e) CALMODULIN-LIKE PROTEINs as input interactors and modulators

An example of massive gene amplification during plant evolution are the calmodulin-like proteins. These are plant-specific Ca^2+^-sensors that likely evolved before calmodulins and are characterized by interacting with specific target proteins ranging from certain heat shock proteins, hubs of the Ca^2+^-dependent protein kinases family, transcription factors, nitric oxide synthases and transporters [49]. In terms of function as modulators of signal integration hubs, they connect input from free Ca^2+^ as second messenger with binding to the mentioned target proteins for activation or inactivation of downstream processes.

**Figure 5.**
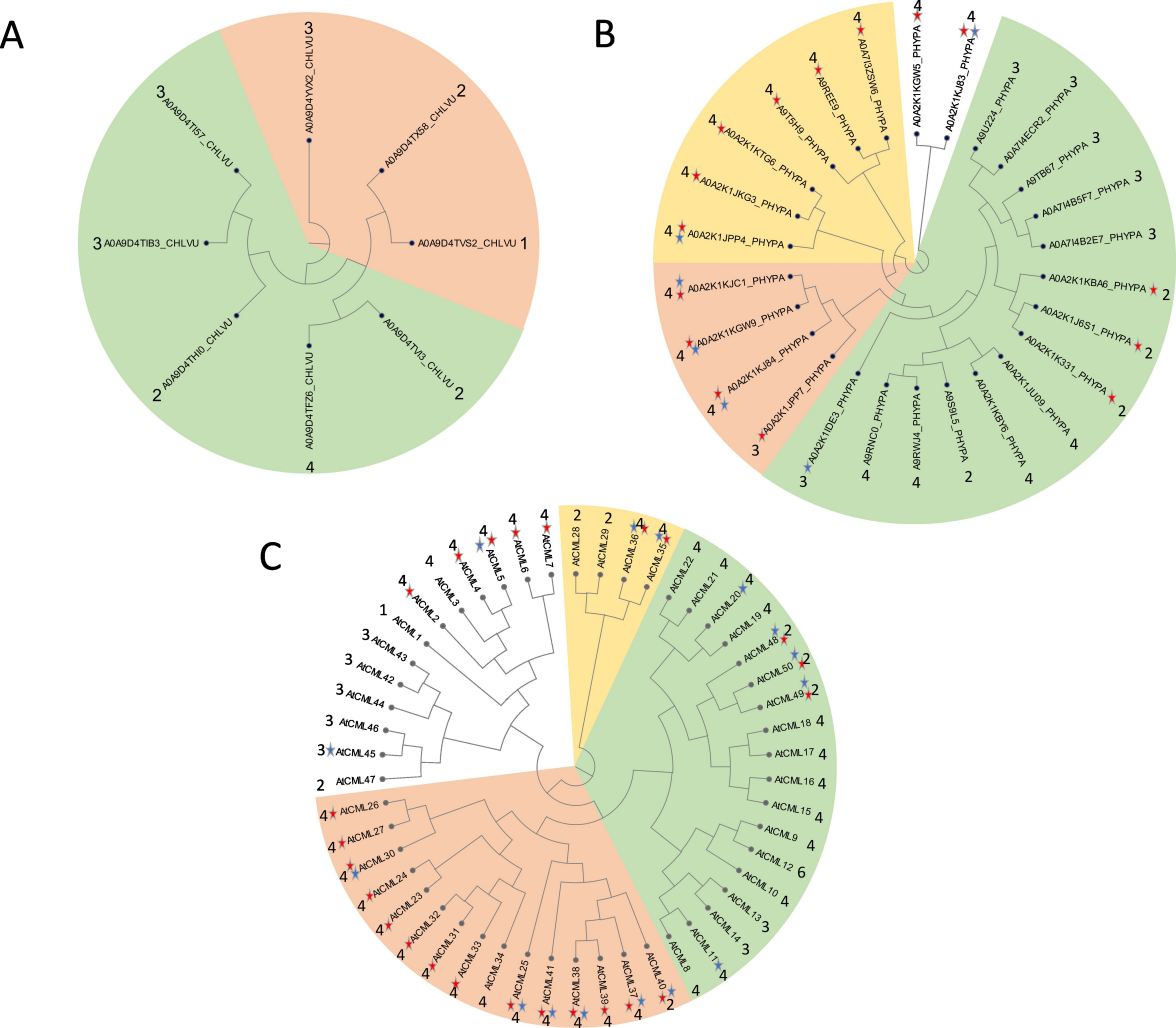
Phylogenetic trees of calmodulin-like proteins in (A) *C. vulgare* (incl. UniProt-ID)*,* (B) *P. patens* (incl. UniProt-ID), and (C) *A. thaliana*. All amino acid sequences were obtained from the UniProt database (9/2024) and used for protein blast in *P. patens* and *C. vulgare*. Red stars mark CMLs with conserved cysteine residues, blue stars mark CMLs with intrinsically disordered regions. The numbers display the number of EF-hands. The presence of domains was obtained from the UniProt database (https://www.uniprot.org/). (See online version for colour references)

During evolution, the number of CMLs increased, displaying 50 isoforms in *A. thaliana* with varying numbers of Ca^2+^-binding EF-hands (ranging from 1 to 6), whereas only 26 and 8 CMLs were identified in *P. patens* and *C. vulgare*, respectively (figure 5). Two characteristic CML features—i.e. conserved cysteine residues, which add redox-regulation capacity to the CMLs, and intrinsically disordered regions (IDRs)—appeared only in *P. patens* and *A. thaliana*, whereas both are missing in *C. vulgare* (figure 5). Some CMLs take over functions like controlling pollen development (CML24) by interfering with actin organization and regulation of ascorbate synthesis (CML10) or they modulate transport processes like CML15 [50–52], but most CMLs link Ca^2+^ to other cellular signalling pathways. For instance, CML43 and CML37 are antagonists in regulating the SA response and are suggested to represent a fine-tuning mechanism of hormonal signalling in *A. thaliana* [53], whereas CML23 and CML24 are involved in Ca^2+^-dependent NO synthesis [54]. There are close relatives of CML43, CML23 and CML24 in *P. patens* (figure 5B), which might carry out similar functions.

CMLs are also involved in biotic stress defence, possibly with high stress specificity—for instance, mediating flagellin-induced callose production to plug plasmodesmata, ethyl vinyl ketone (EVK)-induced calcium pumping and RBOH activation by CML8 [55,56]. Owing to the low distance in the phylogenetic tree, stimulation of RBOH and ACA8 as biotic defence response might be conserved between *P. patens* and *A. thaliana*, too. Last but not least, the IDR-containing CML38 localizes to stress granules under hypoxia, thereby establishing a Ca^2+^-signalling hub in RNA-granules [57]. Similar IDR-containing CMLs were found in *P. patens* as well (figure 5B). Unfortunately, CMLs from *C. vulgare* cluster mostly with uncharacterized proteins from *A. thaliana*, in groups that are characterized by a low number of EF-hands and lack of additional features like IDRs and conserved cysteine residues (figure 5A,C). Whereas organelle-specific isoforms like CML30 and CML3 exist in mitochondria and peroxisomes of *A. thaliana*, respectively [58], the phylogenetic tree provides no hint for organelle-specific isoforms in the other two species (figure 5).

### (f) Approaching network understanding of signal integration

The centrality of a hub is defined by its multiple wiring in the network, allowing for crosstalk. Such crosstalk under diverse environmental conditions allows for specificity, rapidity and plasticity of cellular signals to generate a tailored response and hence regulate energy expenditure towards either defence or growth. From nutrient imbalance-induced K-dependent activation of TOR-signalling to Ca^2+^/ROS-regulated cytosolic K-fluxes under diverse environmental stimuli, which in turn functions as a ‘metabolic switch’ regulating cell fate (programmed cell death (PCD), aerenchyma formation), pollen tube and root hair growth, stomatal movements, etc., the myriad of events associated to K-signalling serves as an ideal example to portray the principal mechanisms of network crosstalk for discussed signalling hubs and Ca^2+^/CMLs/Ca-binding proteins as input elements and modulators. The ideal output results in stress acclimatization and plant survival.

### (g) From hubs to networks, potassium signalling via voltage-gated channels

**Figure 6.**
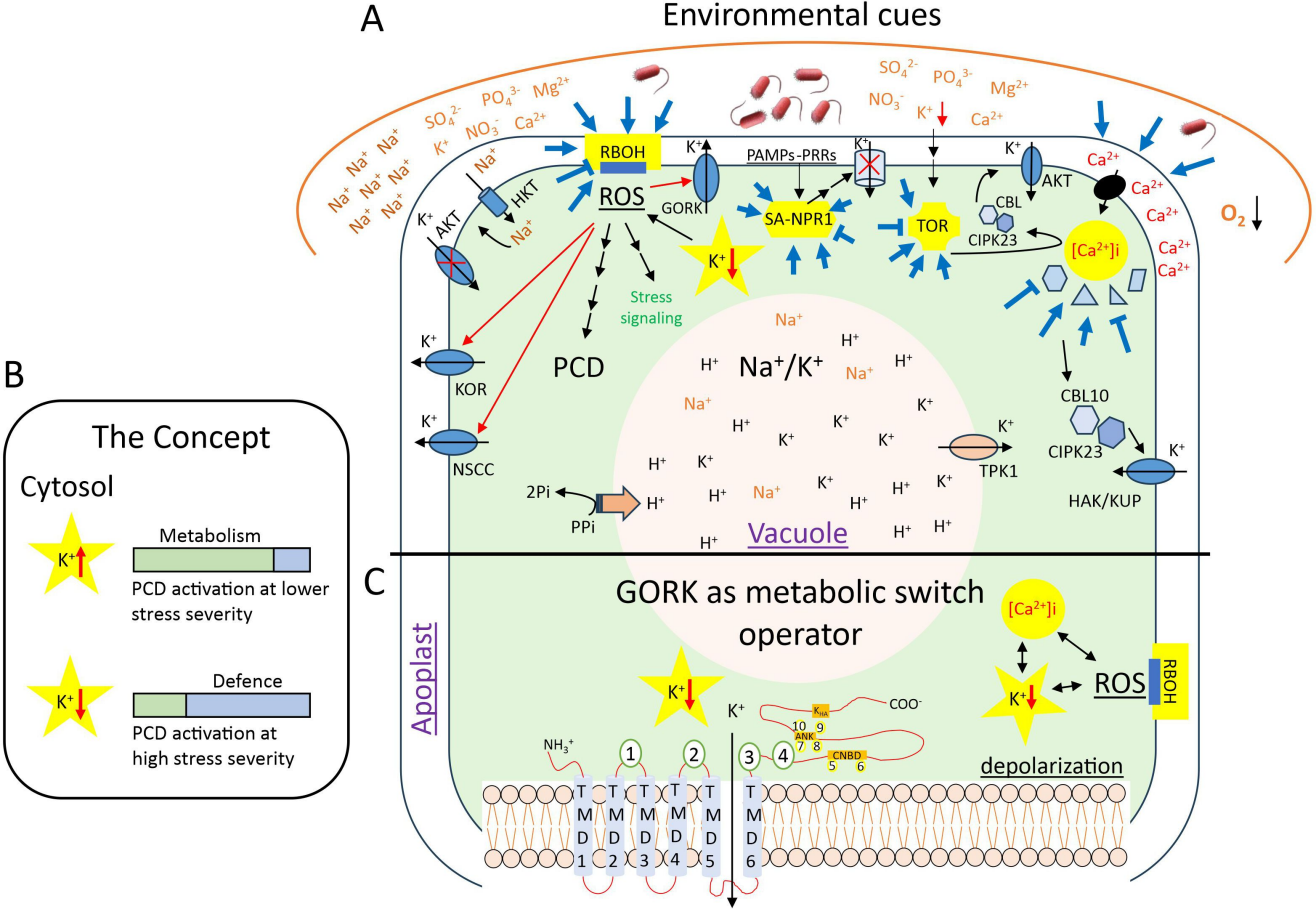
Network crosstalk among discussed signalling hubs feeding into the ‘metabolic switch’ function of cytosolic K^+^ content under diverse environmental cues. Salinity is one of the major abiotic stresses, where besides cellular K/Na ratio, a role of cytosolic K-status as a cellular regulatory mechanism has been identified. The metabolic switching works through activation and deactivation of plasma membrane- or tonoplast-located K-channels. (A) The K-dependent signalling mediates changes in metabolic functions and defence responses concurrent to the established Ca^2+^ or ROS-signatures and contextual with established signalling hubs like TOR or NPR1 (the bold lines ending in stimulation/inhibition of a certain hub depict the already highlighted interactions in figures 2–4). The schematic presents how the discussed hubs (figures 2–4) and modulators could feed into K-signalling, leading to cell fate determination and potential stress acclimatization. The stress acclimatization could be achieved through PCD (e.g. containment of biotrophic pathogen or aerenchyma formation under hypoxia) or stomata closure to prevent further bacterial entry or cellular ionic homeostasis under osmotic challenges, etc. [66–69]. Note: not all known interactions are highlighted. (B) At higher cytosolic K-retention, normal metabolism is favoured, however, as the stress severity slowly increases, the plant cell death will ensue faster as cell runs out of energy available for defence. Stress-induced membrane depolarization results in rapid K-efflux, which—working as a ‘metabolic switch’—helps cells allocate a bigger energy pool for plant defence and results in delayed cell death only at high stress severity [64]. (C) Potential for signal integration has been depicted through gated outward-rectifying K (GORK)-channels, which besides being voltage-gated, also possess multiple binding motifs and domains for different secondary messengers. This highlights the possibilities for them to operate as ligand-gated channels too. For example, besides membrane depolarization and ROS-stimulation, Adem *et al*. [70] identified multiple potential ligands, which are otherwise established to function in stress signalling themselves, that could regulate GORK-activity e.g. (1–4) PIP_2_ (phosphoinositol 4,5-bisphosphate); (5) cAMP; (6) cGMP; (7) GABA (gamma aminobutyric acid); (8) G-proteins (guanine-nucleotide binding proteins); (9) PP−1 (protein phosphatase); (10) ATP. Abbreviations: KOR: K-outward rectifying; NSCC: ROS activated non-selective cation channels; HAK/KUP: high-affinity K⁺ transporters/K-uptake permeases; CBL10: calcineurin B-like protein 10; CIPK23: CBL-interacting protein kinase; TPK1: two-pore K⁺ channels; HKT: high-affinity K-transporter; CNBD: cyclic nucleotide binding domain; ANK: ankyrin domain; TMD1−6: transmembrane domain 1−6; PAMPs: pathogen-associated molecular patterns; PRR: plant pattern recognition receptors; GORK: gated outward-rectifying K-channels.

Potassium (K) is one of the macronutrients required for plant growth. Besides being a structural component, K^+^ performs multiple regulatory functions in different processes like membrane potential, cell turgor, stomatal movement, photosynthesis, pH maintenance and as co-factor for enzymes [59,60]. The advancement in imaging technologies, especially non-invasive ones, has enabled identification of precise cellular ion fluxes including specific K^+^-fluxes, e.g. as markers of pathogen–plant compatibility [61]. Recent studies have ventured into potential for K^+^-fluxes to be considered as critical cellular signals or as signal mediator through its direct and indirect crosstalk with other established signalling molecules like Ca^2+^ (along with diverse Ca^2+^-binding proteins), ROS derived from primary generator systems under abiotic and biotic stresses, e.g. RBOH, or hormones like ABA, JA, auxins, polyamines and SA [62–65] (figure 6A).

In a nutshell, the intracellular K^+^-homeostasis contributes to the regulation of plant metabolism (figure 6B). Therefore, K-signalling has been selected here as an example where the above-discussed hubs feed into and generate diverse stress responses (figure 6A). The multidirectional input through K-influx or -efflux modulation, and diverse ligand-specificities of e.g. GORK-channels, make K-signalling an attractive regulator under complex combinatorial stress situations (figure 6).

K^+^-homeostasis itself is controlled via activation and deactivation of K^+^-selective and non-selective channels and transporters on cell plasma membranes as well as tonoplast. The natural diversity and functionality of the K-channels, especially those in the voltage-gated category, have been explored [71]. From an evolutionary perspective, at least two of the subfamilies of voltage-gated channels, i.e. outward-rectifying K^+^-channels (e.g. GORK in *A. thaliana*; figure 6C) and two-pore cation (TPC) channels, with a prominent function in electrical signalling, have remained conserved during land plant evolution, while all voltage-gated channels share a common origin with the chlorophytes [72]. Further, it is interesting to highlight that the outward-rectifying K^+^-channels evolved independently in the animal kingdom [73].

It has been argued that it is the cytosolic K^+^-content at any given point of time that modulates the plant state between ‘normal’, ‘arrested or hibernated’ under stress to ‘recovery’ upon relieved stress [65]. Therefore, the cytosolic K^+^-content works as a metabolic switch and determines the cell fate under changing environmental conditions (figure 6B) [74]. The metabolic switch is, counterintuitively, defined by rapid K^+^-efflux and not cellular K^+^-retention (figure 6B) [64]. Prominent examples of such regulation have been shown especially for salinity, drought, flooding, heavy metal exposure, biotic challenges and all sources of oxidative stress (figure 6A) [65–68]. Surprisingly, most of the highlighted studies have been carried out in single stress setups, and the specificity of these K⁺-signalling events needs to be identified through testing naturally existing combinations of individual stresses.

### (h) Future perspective

The mechanisms of signal integration discussed in this opinion article have wide applicability. The summarizing schematics given for each example already reveal that many central elements fulfill the criteria of a hub (figures 2B, 3B and 4B). This review did not address the importance of stoichiometry of interactors, and the spatial and temporal coincidence of occurrence as prerequisite for the function as hub. Transcriptome data from tissues or single cells are no reliable proxy of protein amounts, rather we need proteome data to address these points of hub functionality.

One can argue that with increasing sensitivity and coverage of polypeptides by proteomics, many posttranslational modifications will be identified that alter polypeptide functions. Likewise, interactome analyses identify increasing numbers of interacting partners. In the end, all polypeptides might be classified as hubs by just counting interactions and taking a degree of 5 as a lower threshold for this categorization. In the future, the types of interactions and their significance need to be weighed and systematized in order to discover the real hubs of the networks.

Biological networks usually are quite stable and buffered. Responses reach a maximal level and usually do not run away. Robustness and homeostasis rely on local and global feedback mechanisms that dampen the regulation [75]. Although the described examples show features of feedback mechanisms, the precise function and coordination with the feedforward mechanisms often remain qualitative at best. We have poor understanding of the resting and maximally triggered state. The example of NPR1 included a strong role of protein degradation.

Figure 1D,E distinguishes two principal types of integration of different inputs in the output generation of a pathway. The advantage of the hub model (figure 1D) may be derived from metabolic control theory and metabolic pathway regulation [76]. Control of flux through a metabolic pathway usually is shared among the involved enzymes; however, the enzymes either catalysing the committed step or reactions far from thermodynamic equilibrium usually display a higher contribution to the overall control of the pathway. Evolving hubs (figure 1D) instead of multiple regulatory interactions with subsequent pathway elements (figure 1E) may ease balancing the input parameters in a multi-stress environment for optimal tuning of the output.

It will be interesting to apply the hub concept to stress resilience in natural environments that usually pose combinatorial stress conditions to the plants. However, instead opting for strong overexpression or knockdown, the approach should focus on natural or bred variation in amounts or function of the hub protein and its interactions.

## Data Availability

This article has no additional data.
